# Design and Analysis of a Radio-Frequency Moisture Sensor for Grain Based on the Difference Method

**DOI:** 10.3390/mi12060708

**Published:** 2021-06-16

**Authors:** Zhongxu Chen, Wenfu Wu, Jianpeng Dou, Zhe Liu, Kai Chen, Yan Xu

**Affiliations:** Department of Biological and Agricultural Engineering, Jilin University, Changchun 130022, China; czx16@mails.jlu.edu.cn (Z.C.); wwfzlb@126.com (W.W.); doujp@jlu.edu.cn (J.D.); liuzhe20088@jlu.edu.cn (Z.L.); chenkai20@mails.jlu.edu.cn (K.C.)

**Keywords:** grain, moisture, difference method, radio frequency, measuring, accuracy, sensor

## Abstract

Grain moisture is one of the key indexes of grain quality, and acquiring an accurate moisture value is critical for grain storage security. However, the sensors used in the traditional methods for testing grain moisture are based on capacitance, microwave, or radio-frequency methods and still exhibit low accuracy and instability because they are susceptible to the temperature, moisture, and micro gas flow of the air in the granary. In this study, we employed a new design for a radio-frequency moisture sensor for grain. The structure of the sensor is based on the difference method and consists of two parallel probe units. These units are at different distances to the tested grain, resulting in different sensitivities in the moisture measurements. Through a phase difference operation on the test signals, the disturbance variable was reduced. The specific size of the two parallel probes was confirmed by calculation and simulation using High Frequency Structure Simulator (HFSS) software. The simulated and measured parameters of a prototype sensor agreed well. The linear relationship yielded a correlation coefficient of 0.9904, and the average error of the moisture testing was within ±0.3% under the conditions where the VSWR (voltage standing wave ratio) value and return losses were 1.5896 and −20 dB, respectively, at a measured central frequency of 100 MHz. The results indicate that the performance of the sensor was excellent.

## 1. Introduction

Moisture is one of the key values of grain that needs to be controlled during the storage process, owing to its close link with the quality of grain, i.e., grain with high moisture is readily susceptible to microorganisms [1]. With the growth and reproduction of microorganisms, heat and mildew occur in the grain piles, resulting in grain waste. Therefore, the testing of grain moisture is a significant method of securing stored grains [2]. Testing grain moisture in an accurate and stable way is significant for the grain industry.

To date, the use of sensors for grain moisture online testing has been limited owing to commonly occurring shortcomings, such as low accuracy, instability, and a high fault rate. Traditional sensors for grain moisture online testing were designed based on capacitance technology, and there have been some new developments based on technologies, including microwave, radio frequency, nuclear magnetic resonance, and frequency-domain detection [3]. 

The principle of the microwave method for measuring grain moisture is as follows: the working frequency band of microwaves is between 300 MHz and 300 GHz. By using reflected microwaves, the reflection coefficient of the real-time grain moisture can be calculated through the phase change of the reflected waves, and the grain moisture can be determined [4]. As an example, one invasive online microwave method for moisture measurement relies on measuring the phase difference between an incident wave and the reflected wave; a probe emits an electromagnetic wave of 2.45 GHz, forming an incident wave signal, and a reflection is formed when the wave meets the medium [5]. In this design, a single probe, after reinforcement, is used as the detection unit, and the signal source and signal detection circuit are composed of PLL (phase-locked loop) and ALC (automatic level control) technology. 

This technology is in line with the current development trend of instrument detection. However, some key components of the sensors based on this approach, such as a detection probe using a single probe form, readily produce external magnetic field coupling, resulting in electromagnetic interference. Additionally, the microwave band mostly includes UHF (ultrahigh frequency) electromagnetic waves, which necessitates high stability of electronic devices, and instrument failure frequently occurs. The basic principle of the frequency-domain detection method is to convert the analog signal detected by the sensor into a frequency signal to determine the relationship between the frequency signal and the change in grain moisture. 

Usually, this sensor converts a resistance value into a frequency value. This method can detect grain moisture over a wide range; however, the detection resistance value changes with changes in temperature, increasing the detection error [6,7]. The frequency-domain detection method is strongly affected by temperature. The nuclear magnetic resonance method also has certain problems. The basic principle of the nuclear magnetic resonance method can be described as follows: the material absorbs the energy of an electromagnetic field at a specific frequency and a certain temperature (generally 19–35 °C), and then the atoms in the material enter resonance and interact with the external magnetic field of the material; thereby, the properties of the material can be judged by the intensity of the absorbed energy [8]. 

Kanda La and Nelson used the frequency-domain detection method and nuclear magnetic detection method, respectively, to continuously detect grain moisture. The results showed that the 29.9% moisture content of corn decreased from 28.9% (tested at 30 °C) to 25.6% (tested at 10 °C) and from 29.2% (tested at 30 °C) to 27.9% (tested at 10 °C). The research of Kanda La and Nelson showed that the frequency-domain detection method mainly relied on the conversion of the changing resistance value into a changing frequency value to detect grain moisture, while the changing resistance was susceptible to the influence of external temperature and produced a temperature drift; thus, the detection accuracy was easily affected. 

The nuclear magnetic resonance method is dependent on a specific temperature range. Active free atoms excited by radio frequency electrons absorb energy to detect food moisture; however, the detection accuracy is not assured if the temperature is outside of a specific range (19–35 °C). Therefore, temperature changes have a great influence on the detection accuracy of the above two detection methods [9,10]. The radio-frequency method and microwave method have some of the same features, but the working band of electromagnetic radio-frequency waves ranges from 30 MHz to 300 GHz, and the frequency range of radio-frequency waves is generally smaller than that of microwaves. 

For example, a grain moisture detection instrument with a working frequency of 100 MHz was designed using the radio-frequency method; this method was characterized by the use of a three-needle sensor detection probe made of stainless steel. However, in grain detection, especially in granaries, the detection probe is in direct contact with the grain surface, and the grain pressure easily deforms this type of probe, affecting the detection accuracy [11,12]. In addition, when electromagnetic waves are used to detect grain moisture, interference from surrounding environmental factors, such as changes in the magnetic field, temperature, humidity, and metal objects, readily occurs, and under these conditions, this type of method is unable to meet the requirements for the accurate detection of grain moisture.

According to Nelson’s research in 2001, since there is a high correlation between the dielectric properties of grain and the moisture content in grain at any frequency, the dielectric properties of grain at radio frequency are useful for the rapid and nondestructive detection of moisture content. In addition to the moisture content, the dielectric properties of the grains also depend on the frequency, temperature, and bulk density of the granular material. These variables must be taken into account for reliable moisture content detection. 

At radio frequencies below the microwave region, between 1 and 350 MHz, density-independent moisture content determination can be achieved through multifrequency measurements and spectral and statistical data analysis. Density independent moisture sensing can be realized by measuring attenuation and phase shift in a single frequency and using the density-independent function of the dielectric constant and loss factor at microwave frequency. These values can be obtained by any measurement method, including transmission or reflection measurements [13].

In the study of Trabelsi and Nelson (2017). The basic principle of measuring the moisture content in grains and seeds by measuring the dielectric property and the relationship between the dielectric property and the moisture content was introduced. The correlation between the dielectric properties of grains and oil seeds over a wide frequency range (from 1 MHz to 15 GHz) and water content was studied. The effects of the bulk density and temperature of particles and seeds on the dielectric properties were also discussed. At microwave frequencies, the real and imaginary parts of the relative dielectric constant, the dielectric constant, and the loss factor, can be conveniently calibrated using the density-independent moisture function. 

With microwave measurements, it appears that adjusting and correcting the different particle types is not necessary during the calibration process, while temperature can be included in the calibration process. In addition, at microwave frequencies, the error due to the uneven water distribution of corn kernels is less than at frequencies below the microwave range. The detection of moisture in grains and seeds at microwave frequencies is density-independent, which makes monitoring moisture in flowing grains much more successful than low-frequency capacitive sensors. 

Permittivity measurements of grains and seeds using microwave frequencies suggest the possibility of sensing the water content and bulk density of both static and mobile materials simultaneously, providing the moisture content independent of the bulk density. Due to the advantages of high-frequency measurements, the commercial development of new grain and seed moisture meters is expected to improve the reliability and practicability of such instruments in the grain and seed industry [14].

In this study, a method of grain moisture detection based on the difference method and radio-frequency technology was proposed to solve the difficulties of grain moisture detection in existing granaries. In this method, two groups of parallel probes formed a differential structure, and the distance between detection unit 1 and the grain to be tested was greater than that of detection unit 2. Thus, the detection sensitivities of the two groups of probes to the grain moisture to be tested were different, and the signals detected by the two groups of probes were used for a phase difference analysis to obtain the final grain moisture detection signal. 

This differential structure could greatly reduce or even eliminate the temperature drift and interference signals and ensure a stable detection signal. Considering the complex and changeable internal environment of a granary, the detection units were covered on the PCB (printed circuit board) to prevent the deformation of the detection probe in a complex environment (with conditions such as vibration and extrusion), and the detection circuit and detection probes were hidden in the shell to effectively prevent sulfur dioxide and other harmful gases from corroding the probe and grain particles in the granary from causing wear on the probe. HFSS (High Frequency Structure Simulator) simulation and experimental verification showed that the detection error of the differential structure was ±0.3%; this accuracy was better than that of other grain moisture detectors.

## 2. Materials and Methods

### 2.1. Differential Structure and Principles of the Sensor

**Figure 1:**
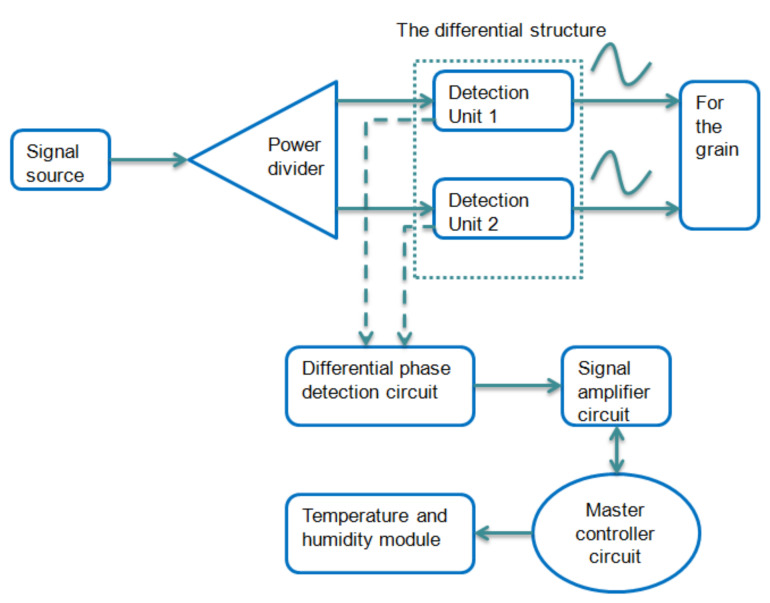
Flowchart of the differential structure of the sensor.

The sensor designed in this paper consisted of the following units: the signal source, power divider, detection units, differential phase detection circuit, amplifier circuit, serial communication circuit, main chip circuit, and temperature and humidity module. Figure 1 shows a working flowchart of the differential structure of the sensor.

**Figure 2:**
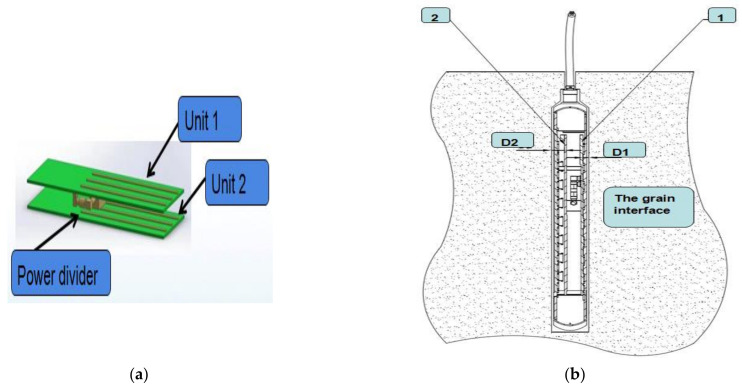
Working state diagram of the differential structure of the sensor: (**a**) Difference structure diagram. (**b**) 1: Detection Unit 1; 2: Detection Unit 2.

Two sets of probes were used in this study. As shown in Figure 2a, the two groups of probes were covered on the PCB. Differential calculations were made for the grain moisture detection signals of the two probe units. This differential structure can minimize the disturbance from the surrounding interference signals. As shown in Figure 2b, the relative distance between detection units 1 and 2 was equal. The distance between detection unit 1 and the grain to be measured was D1, the distance between detection unit 2 and the grain to be measured was D2, and D1 > D2. Detection units 1 and 2 had different sensitivities to the grain moisture content; however, both sets of probes were exposed to the same amount of external interference. 

Therefore, after the phase detection of the two groups of signals, the phase difference between the signals was calculated to obtain the detected analog values. The signal obtained by detection unit 1 was HF1, and the signal obtained by detection unit 2 was HF2. Both HF1 and HF2 included simulated grain moisture contents and simulated external interference signals. Of the sets of probes, unit 1 was close to the grain to be tested, and unit 2 was far from the grain to be measured. The analog signal value of HF1 was $H_{INA}$, and the analog signal value of HF2 was $H_{INB}$. After the calculation of the phase difference, the simulated quantity $V_{HPS}$ was obtained with Equation (1):

(1) $$V_{HPS}=-R_{\emptyset }I_{P}\left[\left|\emptyset \left(H_{INA}\right)-\emptyset \left(H_{INB}\right)\right|-90^{{}^{\circ}}\right],$$

where $H_{INA}$ is the analog value of detection unit 1.$0V\leq H_{INA}\leq 1.8V$; $H_{INB}$ is the analog value of detection unit 2.$0V\leq H_{INB}\leq 1.8V$; $V_{HPS}$ is the simulated quantity obtained after the difference calculation, in units of V; and $R_{\emptyset }I_{P}$ is the phase proportionality coefficient (30 mV/dB).

The phase detector circuit consisted of a SMA RF socket, SMA connector, and SMA adapter. These components were used to connect the probe to the detection circuit, which is equivalent to the coaxial cable connection. The specific QC data were as follows: frequency range: DC—12.4 GHz, voltage standing wave ratio: VSWR ≤ 1.05 + 0.001f, contact resistance: R ≤ 5 MΩ, and insertion loss: IN ≤ 0.15 dB/6 GHz. The phase detector was of the multiplier type; however, the precise phase balance driven by the fully limited signals appears at the outputs of the two logarithmic amplifiers. 

Thus, the phase accuracy measurement was independent of the signal level over a wide range. The AC-coupled input signals could range from −60 dB to 0 dB in a 50 Ω system, from low frequencies up to 2.7 GHz. The outputs provided an accurate measurement of either the gain or loss over a ±30 dB range scaled to 30 mV/dB, and of the phase over a −180°–180° range scaled to 10 mV/degree. The phase and gain output voltages were simultaneously available at loadable ground referenced outputs over a standard output range of 0 to 1.8 V. The output drivers could source or sink up to 8 mA. A loadable, stable reference voltage of 1.8 V was available for precise repositioning of the output range by the moisture detection.

The phase detector circuit worked from −180° to 180°. When the phase detector worked between 0° and 180°, the reference phase angle was 90°. The reference phase angle was −90° when the phase detector work range was 0°~−180°. The slope of the two ranges was the opposite.

**Figure 3:**
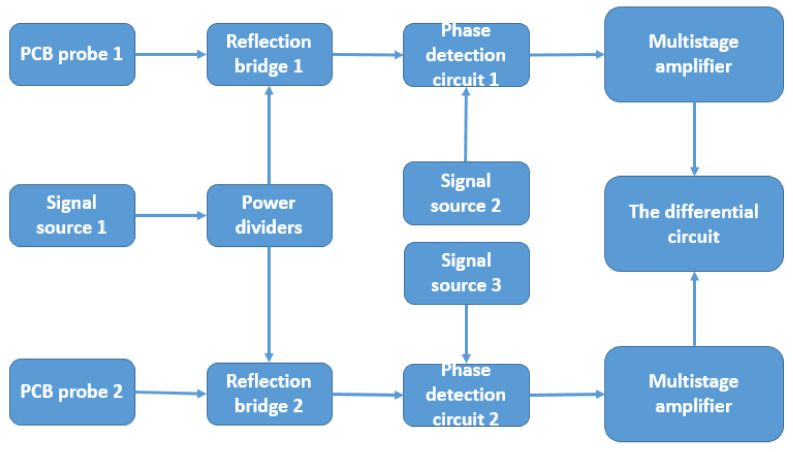
Flowchart of phase detection.

The working principle of phase detection is shown in Figure 3 as the principal block diagram of phase detection. First is the original signal, signal source 1 (A center frequency of 100 MHz. The signal source designed by us was composed of a 100-MHz active crystal oscillator as the main component. Under the working voltage of 3.3 V, a triangular wave of 100 MHz was generated. After modulation and filtering, the triangular wave of a 100-MHz sine wave was generated.), which was divided into two channels by the power divider, and the central frequency point of each signal was 100 MHz. Then, they were transported to the end of reflection bridges 1 and 2, respectively. 

A reflective bridge is a key component for detecting radio frequency signals, which can separate the reflected signal from the incident signal. The principle of the reflective bridge is similar to a Wheatstone Bridge. Compared with directional couplers, the performance of the reflective bridge can fully meet the design requirements with a working frequency of 1–1500 MHz. While the orientation of an ordinary reflective bridge is about 30 dB, this reflective bridge can reach almost 40 dB, which belongs to the standard instrument. The output of reflective bridge 1 was connected with probe 1, and the output of reflective bridge 2 was connected with probe 2. 

Due to the different structure of probes 1 and 2, the distances of the two probes from the measured grain were different, which led to different reflected signals from the two groups. The difference of the two groups of signals could effectively reduce the interference of external factors. The output of the two reflective bridges was connected by the input of the phase detector. Then, signal source 2 (center frequency: 100 MHz) was connected to the input of another phase detector 1, and signal source 3 (center frequency: 100 MHz) was connected to the input of phase detector 2. The special features of the detection circuit design were the reflected signals of the two groups, which differed by multistage phase. 

The reflected signal of probe 1 and the signal of signal source 2 were phase detected, and the same was true for probe 2 and signal source 3. The reflected signal and signal source used for phase detection obtained a more stable signal and solved the problem of weak phase delay. The output of the two phase detectors was connected with a multistage amplifier circuit. The core of the multistage amplifier circuit was an amplifier chip with high accuracy at the mV level. After the precise amplification of the signal of phase detection, the two sets of signals were performed by differential operation through the differential circuit, and stable and clear differential signals were obtained. Finally, the signal after multistage amplification passed through a differential circuit, and a final differential signal was obtained.

In reducing noise interference, especially magnetic field noise interference, many designs have been tested, including a PCB board adopting a four-layer board design, a power network isolated from the signal network, adding a decoupling capacitor to the output of the power supply, separating analog from digital, or the signal source circuit isolated from the main board circuit.

This special phase detection circuit belongs to the multistage phase differential circuit. Although the primary ports of the two sets of signals are phase detected with the signal source, after multistage amplification, the signals obtained through the differential circuit are still in the differential form of the two sets of signals. The signal is particularly stable and can effectively avoid ambient noise and magnetic interference.

The basic theory of this paper was based on the radio-frequency principle. Radio-frequency waves are a type of electromagnetic wave, and their basic propagation characteristics are the same as those of electromagnetic waves. This principle was used in this study. The signal source generated a radio-frequency wave of a certain frequency. This radio-frequency wave was bound to an electromagnetic waveguide as a voltage pulse. 

When the voltage pulse was radiated to a medium through the electromagnetic waveguide, reflection occurred when it met the impedance. The impedance characteristics of the medium could be obtained by comparing the phases of the reflected wave and the incident wave at the receiving end of the signal. According to Skaar C, the dielectric constant of a medium has a certain linear relationship with the water content of the medium. In this paper, the characteristic impedance model of grains with different moisture contents was fitted using the impedance characteristic to achieve grain moisture detection [15,16,17].

### 2.2. Probe Design of the Sensor

The key part of the sensor studied in this paper was the sensor probe of the detection unit. Accurate detection was related to the structure and characteristics of the probe itself. To make the detection signal stable and reduce the power loss of electromagnetic waves, following Heimovaar, the sensor employed a three-needle probe equivalent to a coaxial cable. Compared to other probes, a three-needle probe produces a uniform radiated electromagnetic field, effectively reducing the “skin effect.” 

For example, a five-needle, plate-type structure with signal noise and electromagnetic wave transmission is not stable. According to Robinson and Friedman’s analysis of probe length, especially for a medium with a low dielectric constant (the dielectric constant of grain is 2~4 F/m), when the probe length is 0.05 m, there is a large error in water content detection; therefore, they suggested that the probe should be longer than 0.06 m [18,19,20]. On this basis, Heimovaar formed a mathematical model for the length of the three-needle probe and the water content detected, as shown in Equation (2):

(2) $$\Delta \theta =\frac{d\theta }{d\sqrt{K_{a}L}}\frac{c}{L}\Delta t_{\delta }.$$

In this equation, θ is the volumetric water content, %; $\Delta t_{\delta }$ is the time resolution, s; c is the propagation velocity of an electromagnetic wave, m/s; and L is the length of probe, cm. θ and $K_{a}$ have the following relationship:

$$\frac{d\theta }{d\sqrt{K_{a}}}=0.103.$$

**Figure 4:**
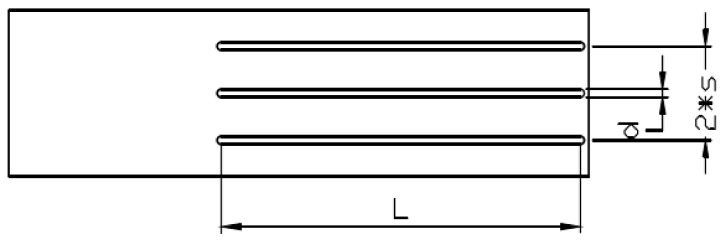
Probe structure size.

As the moisture content of grain is generally not more than 40%, let us say that the value of θ is 0.4. The length *L* of the probe can be calculated as 10.85 cm; therefore, a probe with a length of 10.85 cm was used in this paper. Additionally, according to Knight’s research, d/s>0.1, where d is the probe width and s is the probe spacing. The direction of the electromagnetic field vector around the probe was concentrated, and the magnetic field was highly directional. Therefore, as shown in Figure 4, a detection unit with a width of 3 mm and probe spacing of 15 mm was adopted in this paper [21].

### 2.3. Electromagnetic Field Analysis and Parameter Optimization of the Differential Structure

In this paper, the probe shape, probe size, and differential working principle of the sensor were studied. The basic working principle of the sensor was realized through the radio-frequency pulse wave; a radio-frequency pulse wave is essentially a kind of electromagnetic wave, and electromagnetic waves produce a certain electromagnetic field. Therefore, the analysis of the electromagnetic field was particularly important. Additionally, the performance parameters of the sensor probe were very important to the detection effect. The main working parameters of the probe included the determination of the central working frequency, VSWR (voltage standing wave ratio), S11 (characteristic impedance), magnetic field vector direction, magnetic field radiation energy, etc. The working parameters and structure of the sensor were optimized with HFSS software.

Differential structure of sensor: The main characteristic of this structure is that the two probes are relatively parallel. The reasons are as follows: each group of the structure of the probe was three-pin type; this structure is equivalent to a coaxial cable. A differential structure composed of two “coaxial cables” must have the same length and width, and in the same plane, which is the basic structure of the difference principle. The structure can form a relatively closed and uniform distribution vector electromagnetic field. Therefore, the two groups of probes must be parallel. 

**Figure 5:**
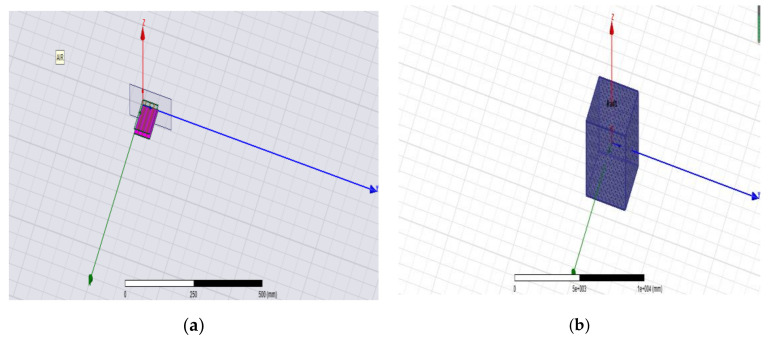
3D simulation model of the probe. (**a**) Differential probe simulation model. (**b**) Radiative boundary model of differential structure.

As shown in Figure 5 below, the 3D model of the probe structure was established using HFSS software. The material of the probe was copper (thickness: 0.1 mm), and the material of the substrate was FR4 (the PCB material). Therefore, the wave port excitation was set as the lumped port excitation. The sweep frequency was set at 95–105 MHz, interpolation was chosen as the sweep type, the number of adaptive iteration steps was 20 to establish the radiation boundary, the dielectric constant of grain was between 2 and 4, and the dielectric constant of the box setting medium was 4. The basic parameter setting was completed, and the model simulation was, thus, carried out.

**Figure 6:**
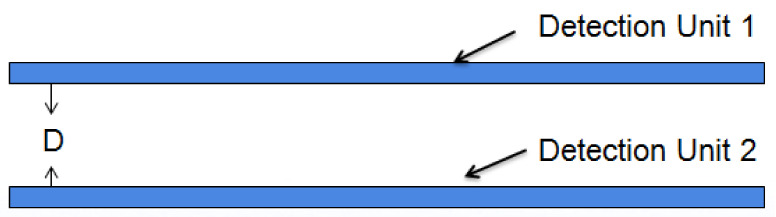
Diagram of the section on the side of the differential structure.

For the special structure studied in this paper, the basic dimensions of the probe detection unit were determined. However, the parallel spacing D of the two sets of probes was not given. As shown in Figure 6, the working magnetic field formed by this structure was relatively complex. The electromagnetic fields generated by the two sets of probes merged into a coupled magnetic field, and HFSS was used to simulate the differential structure and determine the D value.

**Figure 7:**
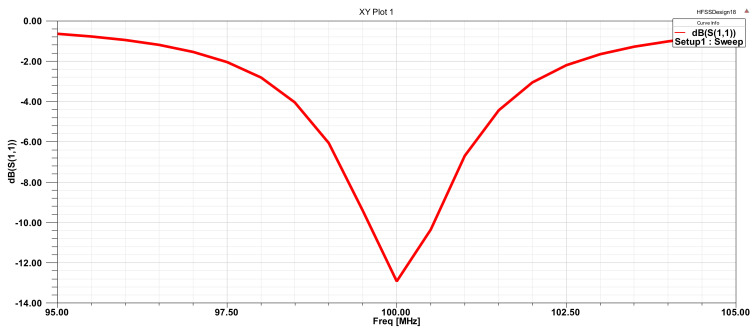
Parametric curve of central frequency point S11.

Determination of center frequency: Before the D value was determined, the frequency of the probe’s operating center, namely, the incidence frequency of the radio-frequency signal source, had to be determined. As shown in Figure 7, at the center frequency of 100 MHz, the corresponding S11 (return loss) parameter was the smallest, at −12.6 dB. Therefore, the center frequency of the radio-frequency signal source was 100 MHz.

**Figure 8:**
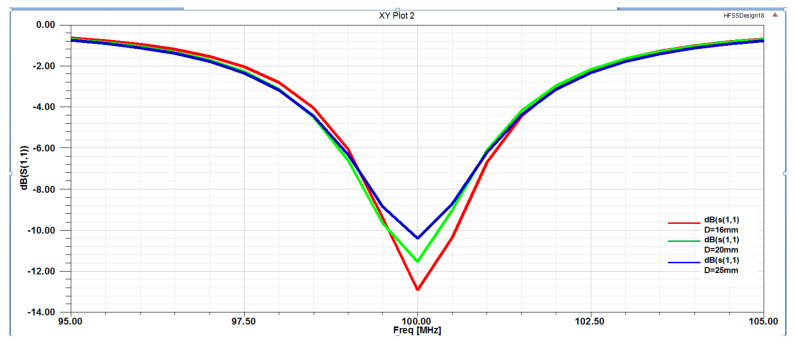
S11 parametric curves with unit spacings of D = 16, 20, and 25 mm.

**Figure 9:**
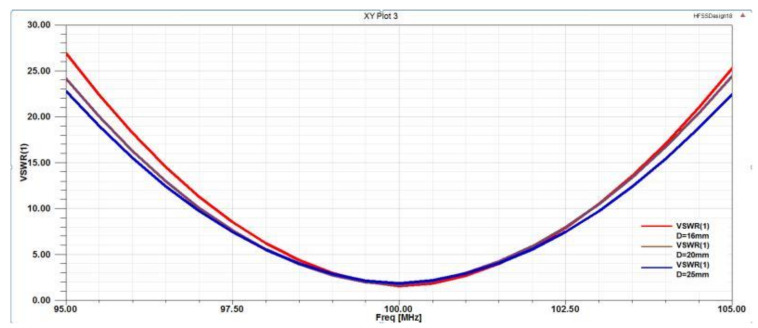
VSWR parameter curves with unit spacings of D = 16, 20, and 25 mm.

Determination of the D value (unit spacing): In Figure 8, at the central frequency point of 100 MHz, the red line is the S11 parametric curve at D = 16 mm, the green line is the S11 parameter curve when D = 20 mm, and the blue line is the S11 parameter curve when D = 25 mm. Three parametric curves for different spacings were compared. When D = 16 mm, the S11 value of the parametric curves was the smallest; S11 = −12.6 dB. At this point, S11 was between 0 and −20 dB. Generally, the closer the S11 parameter was to −20 dB, the smaller the return loss of the probe. Figure 9 shows three VSWR parameter curves with different D spacings. At the center frequency point of 100 MHz, VSWR = 1.5896 when D = 16 mm, VSWR = 1.5901 when D = 20 mm, and VSWR = 1.6025 when D = 25 mm. 

**Figure 10:**
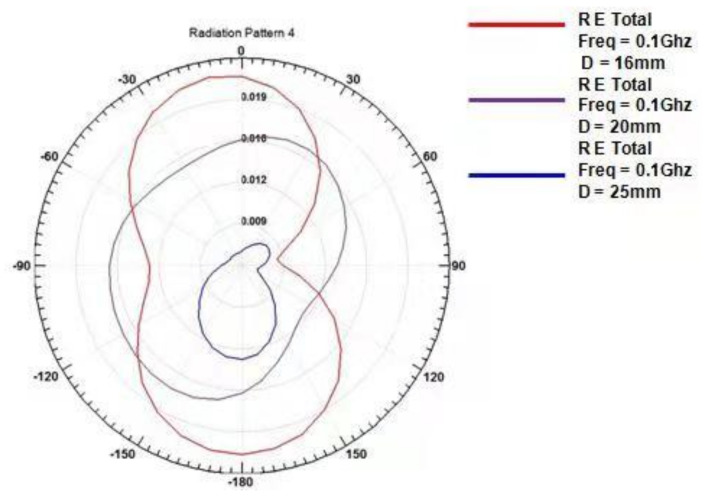
Magnetic field radiation patterns with unit spacings of D = 16, 20, and 25 mm.

**Figure 11:**
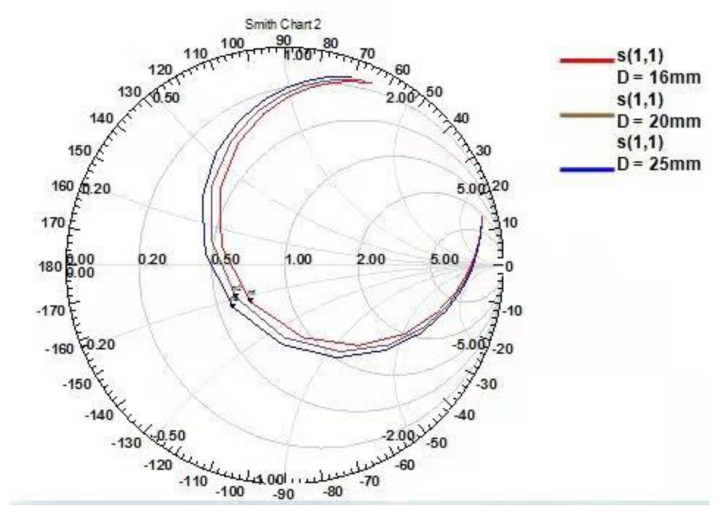
Smith circle charts with unit spacings of D = 16, 20, and 25 mm.

When the value of D was 16 mm, the value of VSWR was the smallest. The closer the VSWR was to 1, the better the voltage standing wave ratio of the probe. Figure 10 shows the magnetic field radiation pattern with three different D spacings. As seen from the figure, when D = 16 mm, the radiation directivity of the probe was good, the radiation angle was greater than 100°, and the radiation path on both sides was unified, reaching 360° balanced radiation. As shown in Figure 11, there were three kinds of Smith circles with different spacings D. When D = 16 mm, compared with other sizes, the red track line was closer to 1, and the normalized impedance was 42.57–0.2387 J Ω, which was converted into a matched impedance of 43.265 Ω. Therefore, this value was closer to the ideal matched impedance of 50 Ω.

Based on the above analysis and the analysis of the probe S11 parameters, VSWR parameters, radiation direction parameters, and Smith circle diagram parameters, we determined that the D spacing between the two sets of differential probes should be 16 mm.

**Figure 12:**
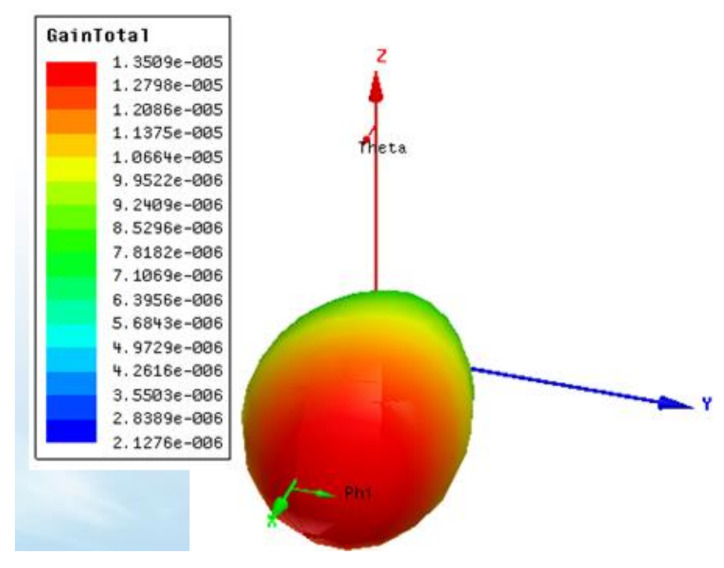
3D magnetic field radiation energy map.

Analysis of the electromagnetic field radiation range: Figure 12 shows the 3D magnetic field radiation energy diagrams. Along the X-axis and Y-O-Z cross section, the red curve reached the maximum value of electromagnetic wave radiation energy, and the main lobe of radiation was wide. At this point, the radiation intensity of the electromagnetic field was 1.3509 × 10^−5^ (A/m). Therefore, according to the Poynting vector surface integral equation [22,23], Equation (3) is as follows:

(3) $$P_{et}=\oint_{S^{\prime }}^{n}S\cdot \bar{n}dS=\oint_{S^{\prime }}^{n}\frac{1}{2}\left(E\times H\right)\bar{n}dS,$$

where $P_{et}$ is the radiation intensity of the surface, *S* is the density of the radiative surface, *n* is the direction coefficient, *E* is the radiated power (design requirement *E* = 1 W), and *H* is the field intensity of the unit vector radiation. After calculation, the maximum radiation surface *S* of the probe can be obtained, and the maximum radiation radius R can be obtained as 0.21435 m.

**Figure 13:**
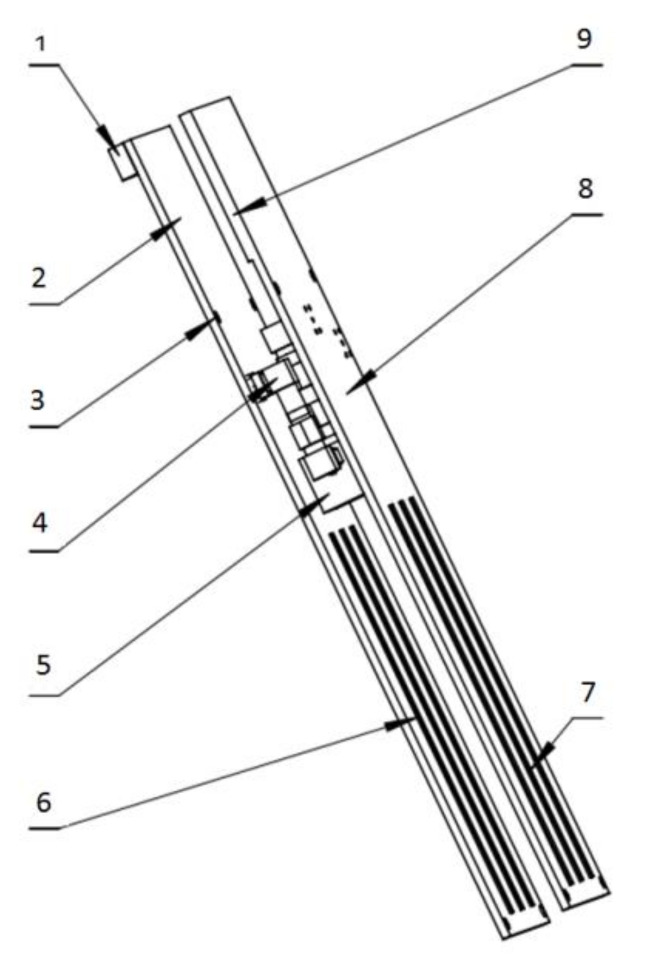
Differential circuit board structure diagram: 1. Temperature and humidity detection module. 2. Signal source circuit. 3. Location hole. 4. The radio frequency (rf) elbow. 5. Power splitter 6. Detection unit 2. 7. Detection unit 1. 8. Circuit board. 9. Main circuit.

**Figure 14:**
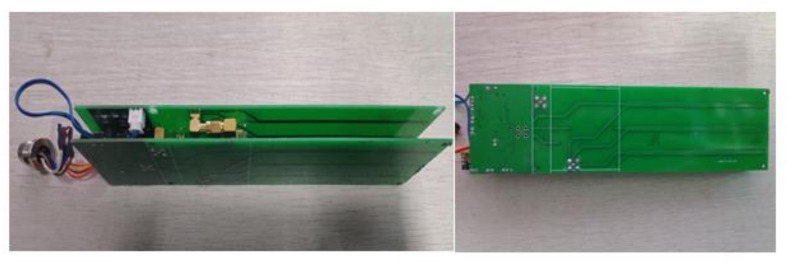
Photograph of the physical differential circuit board.

Based on the above analysis results, the differential structure design of the sensor was completed. The probe was 10.85 cm in length, 3 mm in width, and 15 mm in probe spacing, and the distance between the two sets of detection probes was 16 mm with a differential structure. The structural schematic diagram of the probe units combined with the main circuit to form the circuit hardware, as shown in Figure 13. The physical diagram of the circuit board is shown in Figure 14.

## 3. Test Results and Analysis of the Electromagnetic Field Characteristics of the Probe

**Figure 15:**
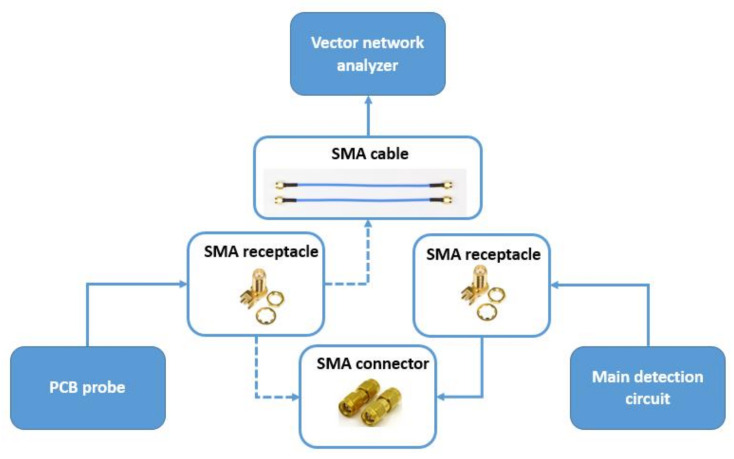
The process of probe testing.

Based on the above theoretical analysis, the differential circuit board of the sensor was designed and completed. To verify the theoretical parameters of the sensor differential structure, in this study, a vector network analyzer was used to detect the parameters of the differential probe. The PCB probe is connected to the circuit via the SMA RF connector. Connecting the SMA RF socket to the network analyzer can isolate the PCB probe from the circuit. The details are shown in Figure 15.

**Figure 16:**
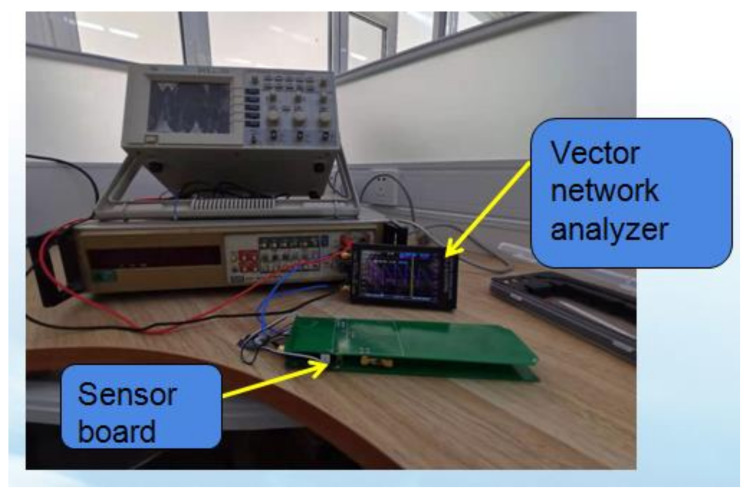
The vector network analyzer test.

The test instrument is shown in Figure 16. A vector network analyzer from the company Nano (Hangzhou, China) was used to detect the center frequency point, S11 parameter, VSWR value, and other parameters of the probe. The data from the detection results and the HFSS simulation results are shown in Table 1. The center frequency detected by the vector network analyzer was 3.6 MHz higher than the simulation result. For S11 parameter detection, the difference was 2.3 dB; the VSWR value difference was 0.1706, and the matched impedance difference was 13.525 Ω. The central frequency points, S11 parameters, VSWR values, and matched impedances detected by the vector network analyzer were close to the simulation results. We concluded that the probe structure of the sensor achieved the desired design result.

The vector network analyzer was used to detect the S21 parameters of the probe, and the input port from the signal source to the phase detector being detected. S21(A) is the signal loss of probe 1, S21(B) is the signal loss of probe 2, and S21A-S21B is the differential signal loss. The details are given in Table 2.

## 4. Calibration of the Grain Moisture Sensor

The calibration of grain moisture sensors is an important step before grain moisture detection and is closely related to the accuracy of sensor detection. The calibration parameters were based on a previous principal analysis. Since the sensor adopted a 100 MHz radio-frequency signal as the signal source and output an analog semaphore after passing the signal through the phase detection circuit and AD conversion circuit, the analog voltage value was adopted as the moisture calibration parameter.

### 4.1. The Calibration Process

According to the balance of grain moisture conditions, low-moisture-content corn (generally below 18%) needs about 120 h at 5 °C, and the balance of the corn moisture content difference should be less than 0.5%. Therefore, before the test, for the sample sealed with film, the temperature was set to 5 °C, and the grain was preserved in a fridge for five days.

The grain moisture in the granary was generally between 13% and 16%; therefore, the calibration test selected grain moisture between 12% and 16%, and the difference in grain moisture was 1%. Five samples with different grain moisture contents were prepared. The initial sample was divided into five equal parts, and the mass of each sample was 4.5 kg. The sample moisture was reduced by heating and drying. The temperature was set to 45 °C, the wind speed was 0.75 m/s, and the sample was weighed every 0.5 h. When the sample weight was equal to the calculated moisture weight, the target moisture value had been reached. The calculation equation is shown in Equation (4):

(4) $$W_{1}=\frac{W_{{}^{\circ}}\left(M_{{}^{\circ}}-1\right)}{M_{1}-1}.$$

**Figure 17:**
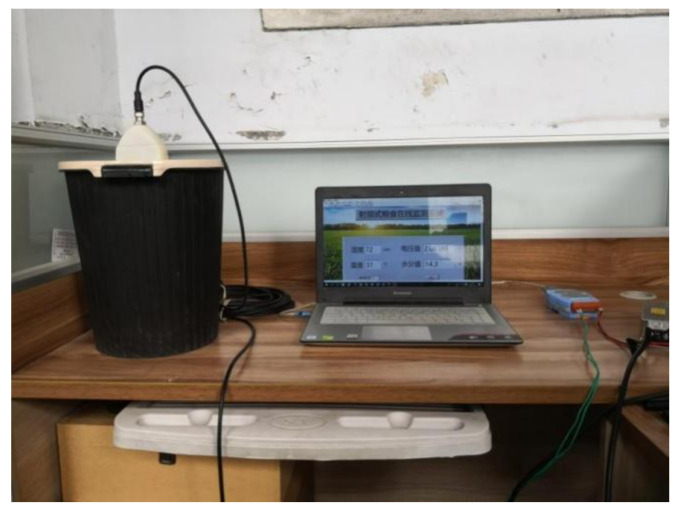
The sensor data acquisition system.

In this equation, $W_{1}$ is the target weight, g; $W_{{}^{\circ}}$ is the initial weight, g; $M_{{}^{\circ}}$ is the original grain moisture, %; and $M_{1}$ is the moisture content of the target grain, %. The prepared sample was tested by the oven method. The sensor was placed vertically in a PVC (polyvinyl chloride) cylinder with a diameter of 500 mm and a length of 0.5 m, and the sample was also placed in the cylinder. The test data collection setup was arranged according to the simulation results of the electromagnetic field in the early stage, imitating the arrangement of grain storage in a granary; the setup is shown in Figure 17. Test data collection parameters are shown in Table 3.

### 4.2. Establishment of a Mathematical Calibration Model

**Figure 18:**
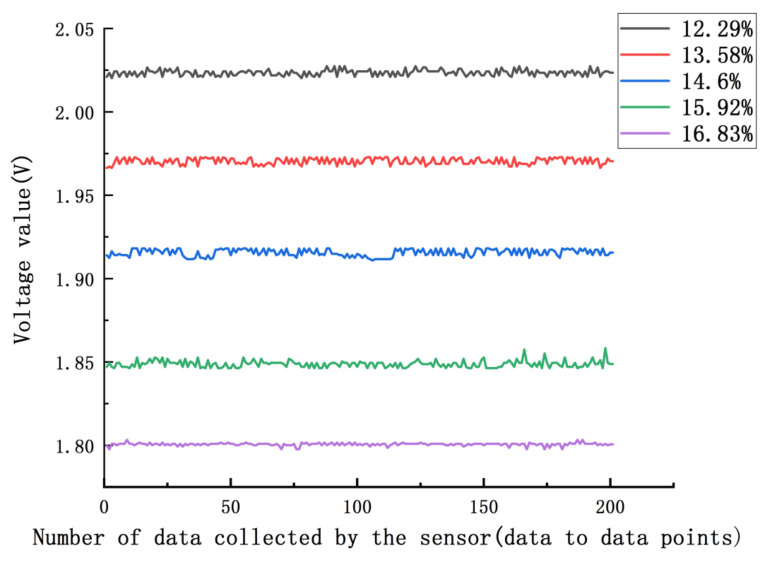
Sensor collection of voltage values.

As shown in Figure 18, for the corresponding voltage values of five samples with different water contents, the voltage values collected were relatively stable overall. The overall degree of differentiation was large; the results after statistical analysis of the data are shown in Table 4. According to the statistical results of the obtained standard deviation and variance, with decreasing water content, the variance and standard deviation of the detected voltage decreased. 

The lower the moisture content of the sample was, the more stable the test voltage. The coefficient of variation decreased with increasing moisture content in the sample, and the discreteness of the detected voltage value tended to stabilize with the increasing moisture content in the sample. The difference between the maximum voltage and the minimum voltage detected for the same grain moisture sample was *p* < 0.0015, which shows that the extreme values of the sample were well distinguished.

According to the skewness analysis in the above table, a linear relationship between the sample moisture content and the corresponding voltage value was established. The linear regression equation of the water content and voltage value of the sample was obtained by the least squares method. Let the deviation between the data *i* and a straight line be:

(5) $$V_{i}=\sqrt{\Delta y_{i}^{2}+\Delta x_{i}^{2}}\;.$$

In the above equation, because the moisture content of individual samples tends to be stable, the change in the moisture content of $\Delta x_{i}^{2}$ is very small, and thus it can be ignored. Therefore, the deviation mainly occurs in the voltage change $\;y_{i}$; therefore,

(6) $$V_{i}=\Delta y_{i}=\left[y_{i}-\left(a+bx_{i}\right)\right].$$

In the above equation, to better optimize the optimal solution of the regression equation, the data points corresponding to coefficients *a* and *b* were determined to be close to a linear line, and thus the smaller the detection error, the smaller the sum of squares of the deviation:

(7) $$\partial \sum_{i=1}^{n}V_{i}^{2}-\sum_{i=1}^{n}{\left(y_{i}-a-bx_{i}\right)}^{2}.$$

In this equation, $\partial \sum_{i=1}^{n}V_{i}^{2}$ is the sum of squares of the total deviation.

The quadratic partial derivatives of Equations (6) and (7) were calculated, and the values of a and b were calculated according to the least squares method to obtain the minimum value. Then, a linear regression equation was obtained. To judge whether the linear correlation equation is correct, it is necessary to test the accuracy and correlation of the regression equation. The accuracy is represented by the remaining standard deviation of $\sigma_{s}$, as shown in Equation (8). The degree of fit between the regression equation and data points is represented, and the correlation is expressed by the variance.

(8) $${\;\sigma }_{s}=\sqrt{\frac{\left(1-R^{2}\right)\left(\sum y_{i}^{2}-\frac{{\left(\sum y_{i}\right)}^{2}}{n}\right)}{n-2}}.$$

Based on the above equation, the calculated values of all parameters are shown in Table 5.

**Figure 19:**
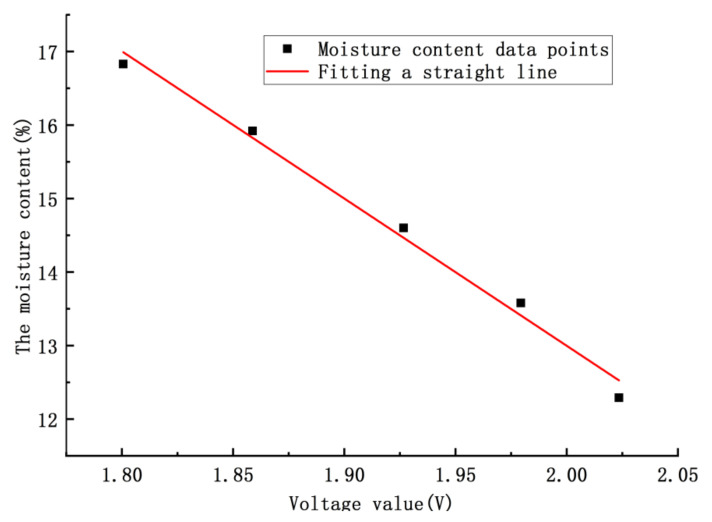
The voltage–moisture characteristic parameter mathematical model.

Table 5 shows that the square of the linear correlation coefficient $R^{2}=0.9904$; thus, the model can be used as a linear regression model for this experiment. Its characteristic parameter regression model curve is shown in Figure 19, and the linear regression model was transformed into:

(9) M=−0.2051×U+0.5.

In the above equation, M is the moisture content of the measured grain and U is the measured voltage.

In Figure 19, according to the fitting linear regression equation, we concluded that the sensitivity of detecting grain moisture was 0.0381 V/1% (the grain moisture content was 1%).

#### 4.2.1. Repeatability Test

The laboratory had maize samples with five moisture levels. The five samples were 20 kg each. The moisture contents of the samples, detected by the oven method, were 11.86%, 13.28%, 14.38%, 15.95%, and 17.11%. Each sample was divided into four equal parts, and each weight was 5 kg. Then, the sensor was placed vertically in the cylinder and the samples were placed. There were four samples in each level, and the number of consecutive collections of each sample was 50. The average value of the collected data of each sample was calculated, and the results are shown in Table 6.

As can be seen from the above table, the detection errors of the sensors are all controlled within ±0.3% for the samples of five levels after repeated detection.

#### 4.2.2. Different Densities for Grain Moisture Detection

The grain density tests were designed as follows: samples of statistical tests were 11.86% (20 kg, 5 kg for each sample, and four samples), 13.28% (20 kg, 5 kg for each sample, and four samples), 14.38% (20 kg, 5 kg for each sample, and four samples), 15.95% (20 kg, 5 kg for each sample, and four samples), and 17.11% (20 kg, 5 kg for each sample, and four samples). The samples were ground into tiny particles successively. The grain moisture of each sample was detected by the sensor, and the number of data points collected from each sample was 50. The average value of the data was sampled and compared with the data detected by the sensor in the statistical test and the data detected by the sensor after grinding, as shown in Table 7.

As can be seen from the above table, the maximum difference between the grain moisture before and after grinding was 0.3224%, and the minimum difference was 0.0005%. Therefore, it can be determined that the size of grain density had little influence on the accuracy of the sensor to detect the grain moisture.

## 5. Simulated Granary Test Analysis of the Sensor

Whether the sensor was suitable for the complex changing environment in an actual granary needed to be further analyzed to build the simulation granary system. A simulation granary test was used to verify the universality of the sensor detection system—namely, to observe whether the sensor could accurately and stably detect grain moisture in a granary for a long time. Whether the accuracy of the sensor in grain moisture detection was affected when the grain moisture migrated in a granary needed to be determined. In the case of mechanical ventilation, whether the sensor could achieve the purpose of accurate detection had to be analyzed.

**Figure 20:**
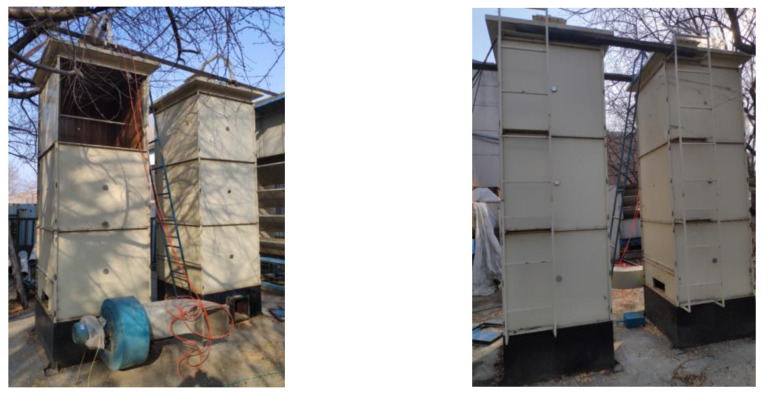
Granary application scene diagram.

A rectangular granary structure was adopted in this experiment. As shown in Figure 20, the existing square bin design, with length × width × height of 1.5 × 1.5 × 4.5 m, was simulated. The whole warehouse was made of steel plates. The inner wall of the warehouse was covered with a 4-cm-thick insulation layer (solid wood and insulation foam). The sampling port was located on the sidewall of the granary, and the four sampling ports were equally spaced 1-m apart. The fan port was connected with a 3-KW fan.

**Figure 21:**
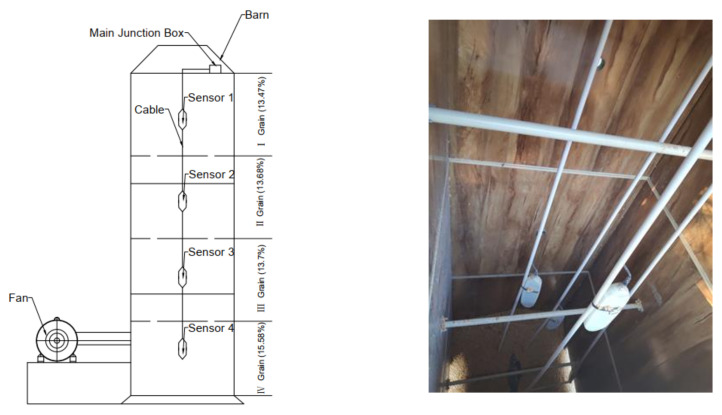
The sensor location layout in the granary and the actual layout in the sensor warehouse.

The sensors were arranged according to the results of the theoretical analysis of the electromagnetic field. As shown in Figure 21, the granary was divided into four floors, and one sensor was placed on each layer. The sensors were placed in the warehouse in a staggered way. This arrangement helped decrease the electromagnetic interference between sensors according to electromagnetic field theory analysis. The grain height of each layer was 0.8 m, and the sensor was fixed on a PPR (polypropylene random copolymer) tube. As shown in Figure 20, the moisture contents of the grain in each layer in the granary, from bottom to top, were 15.58%, 13.7%, 13.68%, and 13.47%. The left side of the granary had a 3-kW fan for mechanical ventilation.

**Figure 22:**
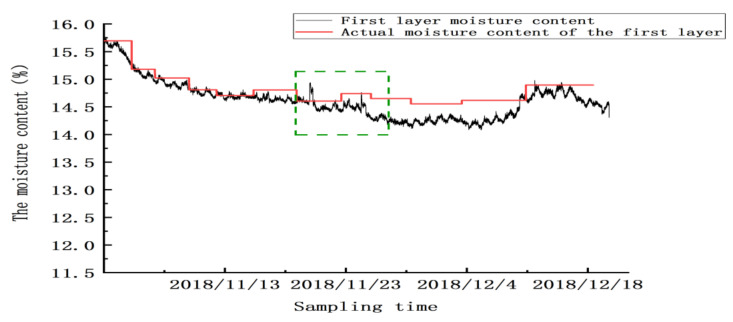
The moisture values of the grain in the first layer.

The experiment was conducted from 4 November 2018 to 20 December 2018. The temperature of the granary varied from −2 to 15 °C, and the humidity varied from 59 to 97 RH%. The experiment was conducted to compare the grain moisture values detected by the four-layer sensor with the actual grain moisture value. As shown in Figure 22, for the first layer, a comparison between the grain moisture value and the actual grain moisture value was made. From 4 to 9 November, the trend of the moisture content detected by the sensor was the same as that of the actual value, and the detection error was ±0.215%. 

**Figure 23:**
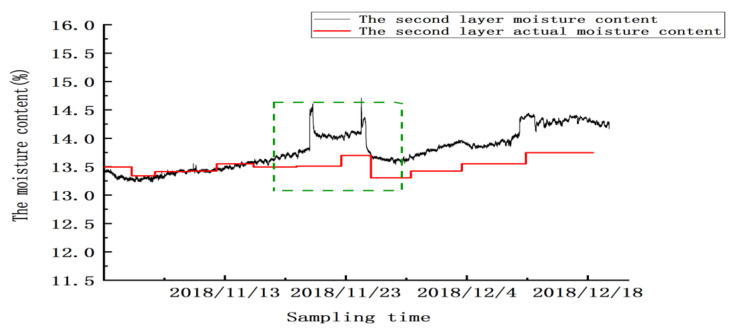
The moisture values of the grain in the second layer.

**Figure 24:**
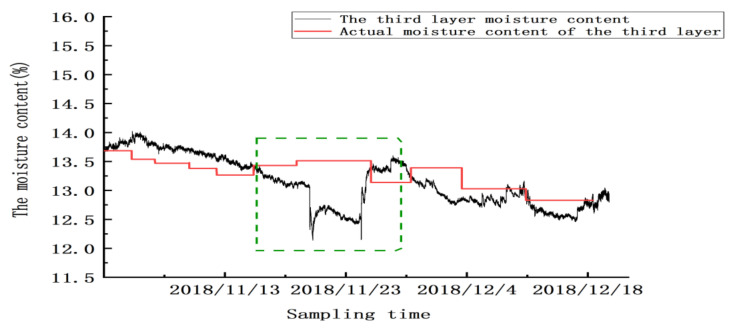
The moisture values of the grain in the third layer.

**Figure 25:**
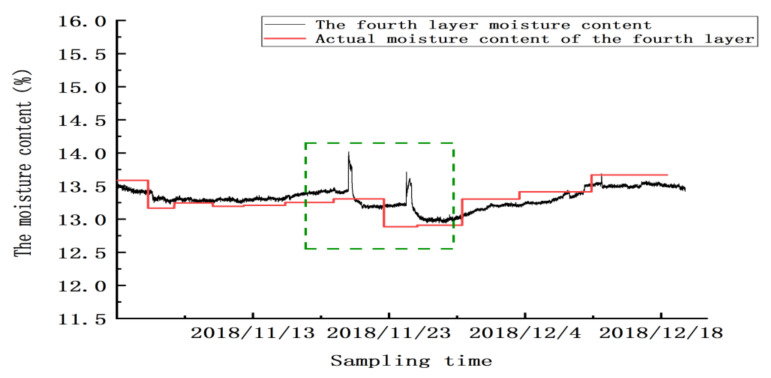
The moisture values of the grain in the fourth layer.

Between 20 and 24 November, there were two abnormal peaks, as shown in the green box in the figure; the abnormality in this area was due to the abnormal test results caused by mechanical ventilation. Twenty-four hours after ventilation, the sensor gradually reached a stable state. From 25 November to 20 December, the sensor’s detection error was ±0.32%, and the mean absolute error for detecting moisture in this layer was ±0.2625% (excluding the abnormal zone value). Similarly, as shown in Figure 23, Figure 24 and Figure 25, abnormal peaks appeared, as highlighted by the green squares, on the second, third, and fourth floors, respectively. In summary, after the abnormal areas were removed, the average error of the sensors in each layer in for detecting moisture was within ±0.3%, as shown in Table 8.

Each technology was used for a specific moisture sensor, and different sensors demonstrated different detection accuracy. There were three parameters of moisture detection by the sensors, as shown in Table 9.

## 6. Conclusions

We proposed and optimized a differential structure for a radio-frequency sensor for grain moisture detection in a granary, and developed a sensor for detecting grain moisture in a granary. The differential structure of the sensor consisted of two groups of detection probes, and the distance D1 between unit 1 and the grain to be measured was greater than that between unit 2 and the grain to be measured. The sensitivities of the two groups of probes to the grain moisture content were different, and the phase difference between the two groups of detection signals HF1 and HF2 was determined to reduce the external disturbance variable. Theoretical analysis and HFSS simulation of the differential structure were carried out. 

After the electromagnetic field characteristic test of the probe, the optimal length (L = 10.85 cm), width (D = 3 mm), spacing of each group of probes (S = 15 mm), and spacing of two parallel probes (D = 16 mm) were determined. When the center frequency of the detection probe with this differential structure was 100 MHz and the minimum return loss was reached, the frequency of the signal source was determined to be 100 MHz. The best matching parameters were optimized, the VSWR value was controlled to 1.5896, and the S11 value was controlled within −20 dB. Combined with the Poynting vector surface integral equation, the radio-frequency radiation region of the differential probe (within the range of radius R 0.21435 m) was calculated. 

Thus, the best matching parameters of the electromagnetic field characteristics of the differential structure were obtained. The sensor was calibrated with the corresponding relationship between the grain moisture content and analog sensor output value; a mathematical calibration model was established, and the linearity of the mathematical model was 0.9904. In simulated granary testing, the results showed that the sensor accurately and stably detected the grain moisture in an actual granary and that the error of the average moisture content detected by the sensor was within ±0.3%.

This radio-frequency moisture sensor based on the difference structure realized the high-precision detection of grain moisture in a granary. However, we found that the accuracy of the sensor in detecting grain moisture was affected to different degrees by the conditions of the ventilated granary in the simulated granary experiment. In future research, the interference factors affecting the sensors in the detection of grain moisture in the actual operation of a granary will be further analyzed to improve the detection accuracy and stability of the sensor, which will provide an effective technical guarantee for the safe storage of grain in a granary.

## Figures and Tables

**Table 1:** Comparison of the simulation results and detection results.

	Center Frequency Point (MHz)	S11 Parameter (dB)	VSWR	Matching Impedance (Ω)
HFSS simulation results	100	−12.6	1.5896	43.265
Vector network analyzer test results	103.6	−14.9	1.765	56.78

**Table 2:** S21 test parameters.

Test Parameters	S21(A) (dB)	S21(B) (dB)	S21A-S21B (dB)
S21	−24.4	−30.5	−21.9

**Table 3:** Test data collection.

Test Date	Grain Moisture	Number of Samplings
21 September 2018	12.29%	200
21 September 2018	13.58%	200
22 September 2018	14.60%	200
22 September 2018	15.92%	200
23 September 2018	16.83%	200

**Table 4:** Data collection and analysis parameters.

Sample Moisture Content	Average (V)	Standard Deviation	Variance	Partial Degrees	Coefficient of Variation	Mean Absolute Deviation	Minimum (V)	Maximum (V)
12.29%	2.0235	0.00177	3.13 × 10^−6^	0.17807	8.74 × 10^−4^	0.00142	2.0202	2.02745
13.58%	1.9704	0.00197	3.88 × 10^−6^	−0.14818	1.00 × 10^−3^	0.0018	1.96638	1.97283
14.60%	1.91554	0.00219	4.78 × 10^−6^	−0.24095	0.00114	0.00196	1.91084	1.91809
15.92%	1.84876	0.00208	4.32 × 10^−6^	1.15695	0.00112	0.0016	1.84628	1.85837
16.83%	1.80062	8.67 × 10^−4^	7.52 × 10^−7^	−1.11308	4.82 × 10^−4^	5.94 × 10^−4^	1.79766	1.8033

**Table 5:** Parameter values.

Parameter	Value
a	−4.876
b	2.628
R^2^	0.9904
$\sigma_{s}$	0.008528

**Table 6:** Repeatability tests.

The Sample Level	Moisture Detection by Sensor (%)			
	Sample No. 1	Sample No. 2	Sample No. 3	Sample No. 4
11.86%	12.1321	11.9822	12.0543	11.9221
13.28%	13.4298	13.1927	13.3433	13.2657
14.38%	14.5213	14.6021	14.2152	14.3729
15.95%	15.7325	15.8894	15.8121	16.0126
17.11%	17.2367	17.1025	16.9879	17.2095

**Table 7:** The density test.

The Sample Level	Moisture Detection by Sensor (%)							
	Sample No. 1		Sample No. 2		Sample No. 3		Sample No. 4	
	Before Grinding	After Grinding	Before Grinding	After Grinding	Before Grinding	After Grinding	Before Grinding	After Grinding
11.86%	12.1321	12.2091	11.9822	12.1325	12.0543	12.1914	11.9221	11.8705
13.28%	13.3298	13.0192	13.1927	13.1922	13.3433	13.2072	13.2657	13.1255
14.38%	14.5213	14.1989	14.6021	14.3202	14.2152	14.4527	14.3729	14.3017
15.95%	15.7325	15.9079	15.8894	15.8703	15.8121	15.9073	16.0126	16.0823
17.11%	17.2367	17.1267	17.1025	17.2199	16.9879	17.0916	17.2095	17.3277

**Table 8:** The average errors of the grain moisture measurements.

Layer Number	Average Error
Layer 1	±0.2625%
Layer 2	±0.2713%
Layer 3	±0.2425%
Layer 4	±0.1317%

**Table 9:** Comparison of the sensor detection parameters of different technologies.

Type of Sensor	Detect the Error	Sensitivity
Parallel plate capacitive	±5%	2.013 F/1% (specific manufacturer)
Single probe microwave type	±4.5%	0.17 V/1% (specific manufacturer)
Differential structure RF	±3%	0.0381 V/1% (this paper)
